# Determining macrophage versus neutrophil contributions to innate immunity using larval zebrafish

**DOI:** 10.1242/dmm.041889

**Published:** 2020-01-17

**Authors:** Emily E. Rosowski

**Affiliations:** Department of Biological Sciences, Clemson University, Clemson, SC 29634, USA

**Keywords:** Innate immunity, Larval zebrafish, Macrophages, Neutrophils

## Abstract

The specific roles of the two major innate immune cell types – neutrophils and macrophages – in response to infection and sterile inflammation are areas of great interest. The larval zebrafish model of innate immunity, and the imaging capabilities it provides, is a source of new research and discoveries in this field. Multiple methods have been developed in larval zebrafish to specifically deplete functional macrophages or neutrophils. Each of these has pros and cons, as well as caveats, that often make it difficult to directly compare results from different studies. The purpose of this Review is to (1) explore the pros, cons and caveats of each of these immune cell-depleted models; (2) highlight and place into a broader context recent key findings on the specific functions of innate immune cells using these models; and (3) explore future directions in which immune cell depletion methods are being expanded.

## Abstract (precis)

**Summary:** Macrophages and neutrophils are distinct innate immune cells with diverse roles in diverse inflammatory contexts. Recent research in larval zebrafish using cell-specific depletion methods has revealed new insights into these cells' functions.

## Introduction

The immune system is best known as a defense against pathogens, but it is also involved in other aspects of human health and disease: wound healing, allergy, auto-immunity, cancer, metabolism, aging and neurological diseases. At a cellular level, this system is composed of two main arms – adaptive immunity (e.g. T cells and B cells) and innate immunity (e.g. macrophages and neutrophils) – as well as components that bridge these arms (e.g. dendritic cells). In order to improve human disease outcomes, it is important to understand the specific functions of these cell types in different inflammatory contexts. Much recent research has focused on the role of adaptive immune cells in human health [most clearly illustrated by the explosion of research on the role of T cells in cancer therapy (Leach et al., 1996; Iwai et al., 2005)], but innate immune cells, including macrophages and neutrophils (Box 1), also play key roles in human health (Nielsen and Schmid, 2017; Coffelt et al., 2016; Kolaczkowska and Kubes, 2013).

**Box 1. Macrophages and neutrophils, the basics**

Macrophages and neutrophils are two major cell types of the innate immune system, the primary function of which is to combat infection. These cells are the primary phagocytic cells, able to take up and destroy both pathogens and cellular debris. However, these cells have a multitude of functions, including secreting cytokines, growth factors, and lipid signaling molecules to orchestrate the behavior of other immune cells. Macrophages can also efferocytose apoptotic cells and promote tissue remodeling, while neutrophils can form neutrophil extracellular traps (NETs) to combat pathogens too large to ingest. Tissue-resident macrophages reside in almost every tissue, ready to respond to any local inflammatory signals. Monocytes can also be recruited from the circulation into inflamed tissue to differentiate into macrophages. Like monocytes, neutrophils primarily reside in circulating blood and are generally the first cells recruited to a source of infection or tissue damage. Both zebrafish macrophages (Torraca et al., 2014) and neutrophils (Henry et al., 2013) are remarkably similar to their mammalian counterparts. Zebrafish macrophages are capable of phagocytosis (Herbomel et al., 1999), pro-inflammatory gene expression and polarization (Nguyen-Chi et al., 2015), and granuloma formation (Davis et al., 2002). Neutrophils have conserved motility mechanisms (Rosowski et al., 2016), and are capable of phagocytosis (Le Guyader et al., 2008) and generating NETs (Palić et al., 2007).

Myeloid cell precursors develop by 12 h postfertilization in zebrafish and functional macrophages and neutrophils are present by 30 h postfertilization (Herbomel et al., 1999; Le Guyader et al., 2008; Lieschke et al., 2002). Both of these cell types derive from a common myeloid progenitor, and their development is dependent on Pu.1 (also known as Spi1b) (Rhodes et al., 2005; Li et al., 2011). While neutrophils only require minimal Pu.1 activity, macrophage development requires early and continual Pu.1, in conjunction with another transcription factor, Irf8 (Tenor et al., 2015; Li et al., 2011; Shiau et al., 2015). Neutrophil development and function is also controlled by colony stimulating factor 3 (Csf3; also known as Gcsf) and its receptor Csf3r (also known as Gcsfr) (Panopoulos and Watowich, 2008).

In zebrafish, these cells are marked by well-established reporters that use cell-specific promoters that are generally different from those used in mice. Macrophages are typically marked by the *mpeg1.1* gene (Ellett et al., 2011); however, other genes are also specific for this cell type, including *csf1ra* (Gray et al., 2011) and *mfap4* (Walton et al., 2015). One caveat is that these markers do not distinguish between tissue-resident macrophages and inflammatory monocytes that can be recruited to sites of inflammation. Neutrophils are typically marked by the *myeloperoxidase* (*mpx*) promoter (Mathias et al., 2006; Renshaw et al., 2006), an enzyme most highly expressed by neutrophils. Other promoters include *lysozyme* (*lyz*) (Kitaguchi et al., 2009), and although this gene may be expressed by macrophages early in development, by 2 days postfertilization, it is specific for neutrophils (Meijer et al., 2008). This finding does highlight another caveat for these markers; while their expression is well studied in the larval stage of zebrafish, it is unknown if they maintain their specificity through the juvenile and adult stages of the animal.

The immune response involves a complex crosstalk between many cells. The clearest way to experimentally define the function of a cell is to deplete that specific cell type in a whole-animal *in vivo* model. Such depletion experiments in mice have contributed major advances on the roles of both macrophages (Hua et al., 2018) and neutrophils (Daley et al., 2008). However, many questions remain unanswered, and murine models have limitations. The larval zebrafish model has emerged as an attractive supplementary model in which to interrogate these questions. The immune system of zebrafish is largely conserved with humans, and, during the larval stage, the adaptive immune system is not yet developed, allowing for the study of innate responses in isolation (Yoder et al., 2002) (Box 2).

**Box 2. The advantages of larval zebrafish**

As an intermediate model, larval zebrafish have many advantages over higher vertebrates. The most highly touted aspect of larvae is that they are relatively small (∼5 mm) and optically transparent, allowing for high-resolution imaging of immune cells throughout an entire live, intact organism. Simple genetic methods utilizing both targeted gene mutation (e.g. CRISPR/Cas9) and exogenous transgene insertion (e.g. Tol2 system) allow experimenters to test the role of specific genes in these responses, even within specific cell populations (Ablain et al., 2015; Zhou et al., 2018) and at specific times (Gerety et al., 2013). More than 100 larvae can be obtained every week from one adult female, allowing for experiments with high statistical power. Larval zebrafish are also ideal for drug screens as small molecules are well absorbed through their skin and inhibitors can be utilized by simply adding them to the larval water (Zon and Peterson, 2005). Adaptive immunity does not become functionally mature until 4-6 weeks postfertilization (Lam et al., 2004), also allowing innate immunity to be studied in isolation in these organisms.

Excellent recent zebrafish innate immunity reviews have focused on findings related to the specific functions of macrophages (Yoshida et al., 2017; Torraca et al., 2014) or neutrophils (Henry et al., 2013; Harvie and Huttenlocher, 2015), or immunity in specific contexts such as infection (Gomes and Mostowy, 2019; Rosowski et al., 2018b; Masud et al., 2017). The purpose of this Review is to provide a broader view of the role of these cell types in diverse biological situations, and to compare and contrast different depletion methods to perhaps explain disparate results and interpretations in the literature. I first briefly discuss mouse models used to study macrophage and neutrophil function (Table 1) and highlight some of the first studies to utilize these models in order to provide historical context. Then, I dive deeper into the larval zebrafish model, first discussing how existing cell depletion methods work, highlighting the most recent findings that were made possible because of these immune cell-depleted models, and exploring their future prospects.

**Table 1.**
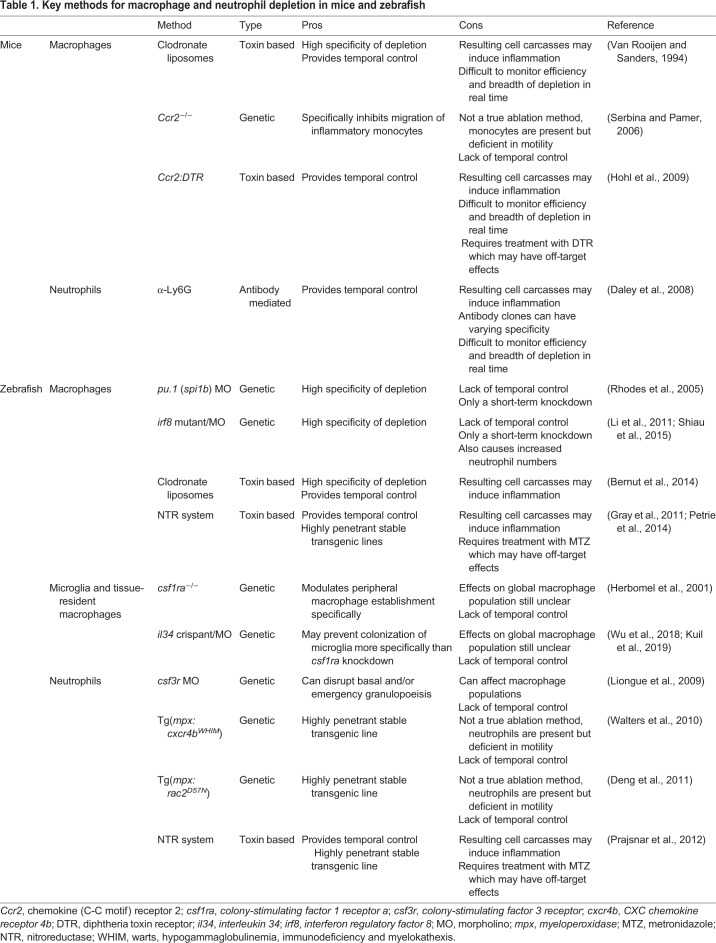
**Key methods for macrophage and neutrophil depletion in mice and zebrafish**

## Innate immune cell depletion in mice

### Macrophages and monocytes

In terms of macrophage function (Box 1), the use of clodronate (see Glossary, Box 3) liposome-mediated depletion has historically identified important roles for macrophages in mice, especially in murine cancer and infection models (Van Rooijen and Sanders, 1994; Moreno, 2018). Macrophages phagocytose these liposomes, releasing clodronate inside the cell, leading to cell death (Frith et al., 1997; Lehenkari et al., 2002). In cancer models, clodronate liposome administration led to decreased tumor growth in multiple studies, demonstrating a role of macrophages in supporting tumor development (Banciu et al., 2008; Zeisberger et al., 2006; Fritz et al., 2014). However, it has become clear that the role and phenotypes of tumor-associated macrophages can vary widely depending on the specific tissue context (Yang et al., 2018; Hobson-Gutierrez and Carmona-Fontaine, 2018). In wound healing, clodronate liposome administration can decrease scarring, suggesting a role of macrophages in fibrosis at a wound (Zhu et al., 2016; Lu et al., 2014).

**Box 3. Glossary**

**Blastema:** a collection of cells competent for growth and regeneration of tissues.

**Ccr2^+^ monocytes:** Chemokine (C-C motif) receptor 2 (Ccr2) is a receptor controlling the recruitment of monocytes out of the blood circulation and into inflamed tissues. Ccr2^+^ monocytes found in tissues are also called inflammatory monocytes.

**CD11b:** subunit of the cell-surface exposed integrin Mac-1. Although found on multiple cell types, including neutrophils, macrophages, and dendritic cells, high expression is often used as a marker for macrophage lineages.

**Chemokines:** a subset of cytokines that specifically modulate migration of cells, especially by attracting immune cells to a source of inflammation.

**Clodronate (also known as dichloromethylene diphosphonate):** an analog of pyrophosphate that is metabolized by cells to create a non-hydrolyzable form of ATP, blocking mitochondrial respiration and leading to cell death. When packaged in liposomes and injected into animals, it is specifically phagocytosed by, and leads to depletion of, macrophages.

**Complement system:** part of innate immunity, this system comprises small, soluble proteins that can be cleaved and activated in a cascade to either directly target pathogen membranes or activate phagocytes.

**Colony-stimulating factor 1 receptor** **(Csf1r; also known as c-fms):** receptor for multiple cytokines [including Colony-stimulating factor-1 (Csf1) and Interleukin 34 (Il34)] that has effects on the production, differentiation, migration and activity of macrophages. Zebrafish have two copies of the gene: *csf1ra* and c*sf1rb*.

**Colony-stimulating factor 3 [Csf3;** **also known as Granulocyte colony-stimulating factor (GCSF)]:** a cytokine that stimulates new neutrophil production from the hematopoietic tissue and their release into the blood.

**Crispant:** an F0 embryo or larvae that was injected with Cas9 protein and guide RNAs (gRNAs) for CRISPR-based genome editing during the very earliest developmental stages (1-4 cells). Depending on the efficiency of the Cas9/gRNA, crispants can have stable mutations at the target locus in the majority of cells, but are genetically mosaic at this locus.

***Cryptococcus neoformans*:** an opportunistic fungal pathogen primarily of immunosuppressed individuals, especially HIV/AIDS patients. Infection in healthy individuals is rare. It grows as a haploid yeast form, but can also undergo mating and meiosis to produce spores that germinate into yeast.

**CXC chemokine receptor-1/2 (Cxcr1/2):** the primary chemokine receptors controlling neutrophil migration to sites of inflammation. In humans and zebrafish, the primary ligand for Cxcr1/2 is Chemokine (C-X-C motif) ligand 8 (also known as Interleukin-8). Mice lack Cxcl8, and Cxcl1/2 can bind to this receptor instead and control neutrophil migration.

**Cytokines:** small, usually secreted proteins that affect the activation and behavior of cells, especially immune cells.

**Diphtheria toxin receptor (DTR):** this receptor can be used to target and kill specific cell populations in a time-controlled manner. The receptor is exogenously expressed under a cell-type specific promoter, and, when diphtheria toxin is administered, only DTR-expressing cells are affected and killed.

**Emergency granulopoiesis:** a response to infection or inflammatory stimuli that results in increased production of neutrophils from the hematopoietic tissue.

**Gr-1:** a marker/antigen originally thought to be specific for neutrophils but that includes epitopes from both Ly6G and Ly6C, and is therefore found on both neutrophils and monocytes.

**Interleukin 1 beta (Il1b):** a major pro-inflammatory cytokine. Requires processing by caspase enzymes to cleave off an inhibitory pro domain before it can be secreted and active. Binds to the Interleukin 1 receptor (Il1r).

**Lipopolysaccharide (LPS):** molecules that are a fundamental component of the outer membrane of the cell wall of Gram-negative bacteria. Recognized by Toll-like receptor 4 (TLR4) in humans and mice.

**Lysozyme M (LysM):** antimicrobial enzyme that can degrade peptidoglycan, a major component of Gram-positive bacterial cell walls. Can be expressed by multiple myeloid lineages, but is often used as a marker for neutrophils.

**Ly6C:** cell surface protein, expressed on both neutrophils and monocyte/macrophage lineages. A previously used antibody clone used to deplete neutrophils in mice (RB6-8C5) was found to cross-react with this target, leading to unintended depletion of monocytes and macrophages.

**Ly6G:** cell surface protein, expressed primarily on neutrophils. Targeted by the antibody clone 1A8 to specifically deplete neutrophils.

**Mechanosensory hair cell:** sensory receptor cells that contain membrane channels that open in response to mechanical stimulation. In humans and mice, these are found in the auditory system responding to sound vibration; in fish, they can be found in the lateral line to detect movement in the surrounding water.

**Morpholino:** antisense oligonucleotides made from synthetic, stabilized nucleic acids. Used to inhibit protein expression either by directly blocking translational initiation or by blocking mRNA splice sites, leading to mis-splicing, and inclusion of introns or exclusion of exons.

**Myeloid cells:** cells that arise from a common myeloid progenitor in the hematopoietic tissue, including neutrophils, basophils, eosinophils, mast cells, monocytes, macrophages and some dendritic cells.

**Parabiosis:** the joining of two separate individual animals such that they share a circulatory system and can exchange cells through the blood flow.

**Synteny:** conservation of the physical architecture of the genome. For example, the existence of similar blocks of genes in similar positions in multiple organisms.

**Tumor necrosis factor alpha (Tnfa):** a major pro-inflammatory cytokine. Signals through the TNF receptors Tnfr1 and Tnfr2, leading to activation of the NF-κB transcription factor and MAP kinase pathways.

**GAL4-UAS system:** GAL4 is a yeast-derived protein that binds to upstream activation sequence (UAS) enhancers and activates transcription of genes downstream. This allows for construction of genetic lines with promoter-specific expression of gene targets where these two pieces (promoter:GAL4; UAS: gene target) are separable and interchangeable.

**Vascular endothelial growth factor A (Vegfa):** a growth factor that targets endothelial cells, promoting vascular permeability, angiogenesis and cell migration.

In the context of infection, clodronate depletion experiments in mice revealed that macrophages are required for a successful immune response against multiple pathogens. For example, during infection with viruses [e.g. Herpes simplex virus (Pinto et al., 1991)], bacteria [e.g. *Pseudomonas aeruginosa* (Kooguchi et al., 1998; Manicone et al., 2009), *Listeria monocytogenes* (Pinto et al., 1991), *Klebsiella pneumoniae* (Cheung et al., 2000)] and fungi [e.g. *Candida albicans* (Qian et al., 1994), *Aspergillus fumigatus* (Bhatia et al., 2011)], macrophage depletion can lead to decreased mouse survival and/or increased infectious burden. However, other conflicting studies report that macrophages are not required for immunity to some of these same pathogens, including both bacteria [e.g. *P. aeruginosa* (Koh et al., 2009; Cheung et al., 2000)] and fungi [e.g. *A. fumigatus* (Mircescu et al., 2009)]. One possible variable in these studies is the method of liposome administration. Clodronate liposomes can be injected intravenously to systemically deplete macrophages. However, they can also be administered locally to deplete cells in just one organ such as the lungs (Cheung et al., 2000; Kooguchi et al., 1998). Independent of the administration route and target, macrophage depletion is difficult to monitor in multiple tissues in an intact, live mouse.

Genetic models for macrophage depletion in mice have recently been reviewed elsewhere (Hua et al., 2018), and here we just provide a brief overview of the most commonly used of these methods. Specific cells can be depleted with a diphtheria toxin receptor (DTR; Box 3) system (Saito et al., 2001). DTR expression is controlled by a specific promoter and, upon diphtheria toxin administration, it leads to the death of DTR^+^ cells. This system has been used with both *LysM* and *CD11b* (also known as *Itgam*; Box 3) promoters. Although LysM and CD11b are expressed on multiple myeloid lineages (Gordon et al., 1974; Keshav et al., 1991; Faust et al., 2000; Dziennis et al., 1995), DTR-mediated ablation of cells expressing these markers only seems to target macrophages, not neutrophils (Duffield et al., 2005; Goren et al., 2009). Both *LysM:DTR* and *CD11b:DTR* mice were used to study macrophage function in response to wounding, finding that macrophages promote wound healing, primarily at the early stages of wound response (Mirza et al., 2009; Lucas et al., 2010).

Ccr2^+^ monocytes (Box 3) can also be specifically depleted with the DTR system (Hohl et al., 2009). Ccr2 is required for monocyte infiltration to an infection site, and *Ccr2*^−/−^ mice are also used to interrogate the function of these cells at sites of inflammation (Serbina and Pamer, 2006). Using DTR-mediated depletion, inflammatory monocytes were found to be important for clearance of both bacterial [e.g. *L. monocytogenes* (Kurihara et al., 1997), *Mycobacteria tuberculosis* (Peters et al., 2001; Scott and Flynn, 2002)] and fungal [e.g. *A. fumigatus* (Hohl et al., 2009), *C. albicans* (Ngo et al., 2014)] infections. Ultimately, the most complete information can be gleaned from using multiple depletion models in conjunction, as illustrated by a recent study on the role of macrophages in the response to Vaccinia virus, which used a variety of methods, including systemic and local clodronate administration, *LysM:DTR* and *Ccr2*^−/−^, to conclude that local and systemic macrophage populations have different functions in the control of viral replication and dissemination (Davies et al., 2017).

### Neutrophils

The primary method for depleting neutrophils in mice is administration of an antibody targeting Ly6G (also known as Gr-1; Box 3), first done primarily with the RB6-8C5 monoclonal antibody (Tepper et al., 1992; Conlan and North, 1994). The mechanism of this depletion is not fully understood, but seems to depend on the presence of macrophages (Bruhn et al., 2016). The RB6-8C5 antibody clone was used extensively to determine the function of neutrophils in multiple inflammatory contexts, including infection, wounding and cancer, as detailed below.

In response to infection, such studies found that neutrophils protect the host against a large range of pathogens, including bacteria [e.g. *Listeria monocytogenes*, *Salmonella typhimurium*, *Yersinia enterocolitica* (Conlan, 1997), *P. aeruginosa* (Koh et al., 2009), *Staphylococcus aureus* (Mölne et al., 2000)] and fungi [e.g. *C. albicans* (Romani et al., 1997), *A. fumigatus* (Stephens-Romero et al., 2005)]. However, excess neutrophil function was also found to harm the host, most likely due to excess tissue damage (Gresham et al., 2000; Romani et al., 1997). In the context of wounding, the role of neutrophils depended on the age of the mice, with neutrophils promoting wound repair in older mice but having little effect in young mice (Nishio et al., 2008). The role of neutrophils in cancer also varied in studies using the RB6-8C5 antibody. Neutrophils were found to promote growth (Pekarek et al., 1995; Seung et al., 1995) and metastasis (Tazawa et al., 2003; Spicer et al., 2012) of injected cancer cells. However, in an experiment in which the injected cancer cells were engineered to express colony-stimulating factor 3 (Csf3; also known as GCSF; Box 3), neutrophils could promote tumor regression (Stoppacciaro et al., 1993).

However, although the RB6-8C5 antibody clone was chosen to target Ly6G, it was later found to cross-react with Ly6C (Box 3), a marker that is also expressed on macrophages and monocytes, and thus this antibody can also deplete subsets of these cells (Daley et al., 2008; Lee et al., 2013). A different monoclonal antibody (1A8), which was originally less commonly used, is more specific for neutrophil depletion, targeting Ly6G but not Ly6C (Daley et al., 2008), but this issue highlights the difficulty in developing truly specific immune cell depletion methods.

## Immune cell depletion in larval zebrafish: how they work, pros, cons and caveats

Overall, only a few systems for specific and reproducible depletion of innate immune cell types are available in mice, and experiments are complicated by the difficulty of monitoring cell depletion globally in the animal. As immune cells can be easily monitored in the entire living organism through microscopy, the larval zebrafish emerged as a popular model for the study of innate immune cells *in vivo* (Levraud et al., 2008) (Box 2).

Zebrafish have both innate and adaptive immune systems that are similar to those of mammals, including the key cell types (e.g. T and B cells, macrophages, neutrophils, eosinophils and natural killer cells), cytokines (Box 3; e.g. TNF, IFNs, IL-10, IL-12 and TGFβ), receptors (e.g. TLRs) and soluble factors [e.g. complement system and antibodies (van der Sar et al., 2004; Renshaw and Trede, 2012)]. The genome of zebrafish is also relatively conserved with humans: 70% of human genes have an ortholog in zebrafish (Howe et al., 2013), and this conservation is often also accompanied by significant synteny (Box 3) (Barbazuk et al., 2000). While genome duplication in teleost fishes contributed to the existence of multiple copies of some human gene orthologs (Glasauer and Neuhauss, 2014), zebrafish carry orthologs for 84% of human disease-associated genes (Howe et al., 2013).

The function of innate immune cells and pathways can be studied in isolation in larval-stage zebrafish. Adaptive immunity does not functionally mature until 4-6 weeks postfertilization (Lam et al., 2004), while myeloid cell (Box 3) precursors develop by 12 h postfertilization (Lieschke et al., 2002). Functional macrophages and neutrophils are present by 30 h postfertilization (Herbomel et al., 1999; Le Guyader et al., 2008). Their small size, optical transparency and the existence of reliable markers for both neutrophils and macrophages (Box 1) also allow for monitoring depletion of both the cell type of interest and the possible off-target cells in the entire larvae (Ellett et al., 2011; Mathias et al., 2006; Renshaw et al., 2006; Walton et al., 2015). The simplicity of genetic manipulation in zebrafish has promoted the rapid increase in available models in which these cell types are depleted, and findings can now be validated with multiple depletion strategies.

**Fig. 1.**
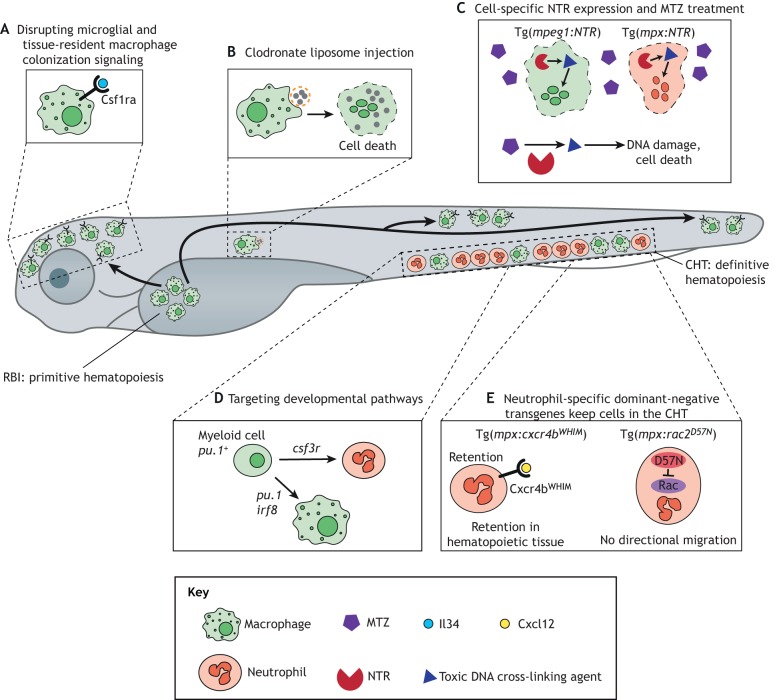
**Immune cell depletion methods in larval zebrafish.** Multiple techniques can be used in larval zebrafish to modulate numbers of macrophages and neutrophils. (A) Primitive macrophages that develop in the RBI seed peripheral tissues, especially the brain to form microglia, via expression of the Csf1ra receptor and recognition of Il34 produced in tissues. Genetically targeting either of the genes encoding these proteins can abolish this seeding and prevent the development of microglia. (B) Injected clodronate liposomes are taken up specifically by macrophages, leading to macrophage cell death and ablation. (C) NTR can be transgenically expressed in a specific cell type and, upon treatment with MTZ, the drug is converted to a toxic compound, leading to cell death, specifically in NTR-expressing cells. (D) Genes that are required for differentiation from progenitor cells (*pu.1* or *irf8* in macrophages, *csf3r* in neutrophils) can be targeted genetically to prevent the development of these cells. (E) Dominant-negative forms of proteins with roles in neutrophil motility and release from CHT are expressed under a neutrophil-specific promoter, preventing these cells from migrating to sites of inflammation. CHT, caudal hematopoietic tissue; Csf1ra, colony-stimulating factor 1 receptor a; *csf3r*, *colony-stimulating factor 3 receptor*; Cxcl12, CXC motif chemokine ligand 12; Cxcr4b, CXC chemokine receptor 4b; Il34, interleukin 34; *irf8*, *interferon regulatory factor 8*; *mpx*, *myeloperoxidase*; MTZ, metronidazole; NTR, nitroreductase; RBI, rostral blood island; WHIM, warts, hypogammaglobulinemia, immunodeficiency and myelokathexis.

**Fig. 2.**
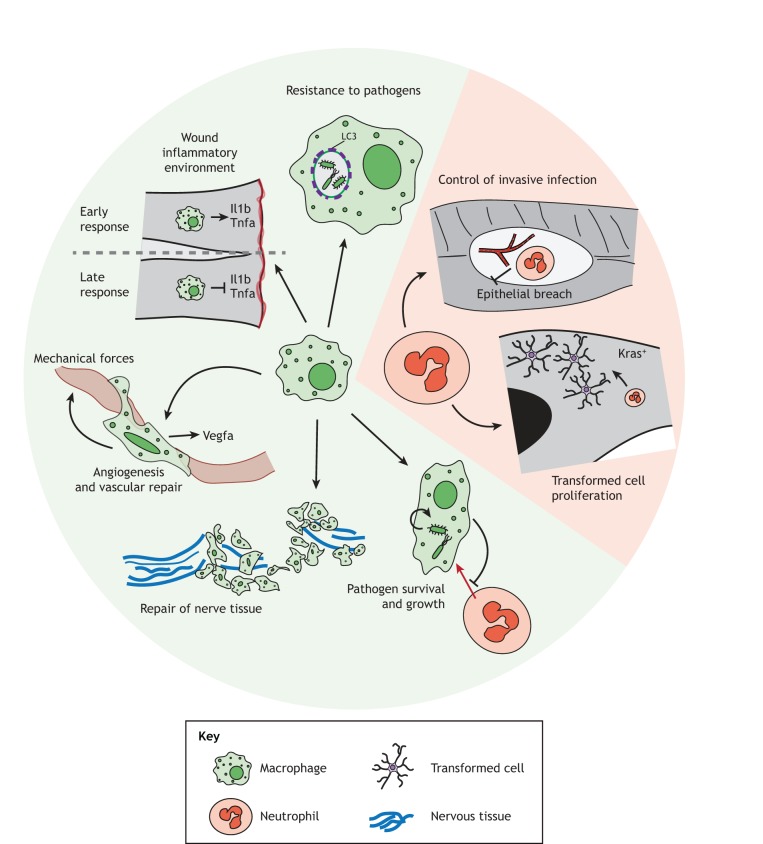
**Recent key advances on cell-specific innate immune functions in larval zebrafish.** Recent studies have highlighted many different roles for macrophages and neutrophils in a range of immune contexts. In response to pathogens, macrophages can target and kill these microbes; for example, in response to the bacterial pathogen *Salmonella*, macrophages use LC3-associated phagocytosis to control this pathogen (Masud et al., 2019). But, in other infections, such as the bacterial pathogen *B. cenocepacia* (Mesureur et al., 2017) or the fungal pathogens *A. fumigatus* (Rosowski et al., 2018a), *T. marneffei* (Ellett et al., 2018) or the *C. neoformans* spore form (Davis et al., 2016), macrophages actually provide a protective niche for pathogen survival and growth. In sterile wounding conditions, macrophages can modulate the inflammatory microenvironment (Tsarouchas et al., 2018; Hasegawa et al., 2017; Nguyen-Chi et al., 2017), use Vegfa activation and mechanical forces to promote angiogenesis and vascular repair (Liu et al., 2016; Gurevich et al., 2018), and promote the repair and regrowth of damaged nerve tissue (Carrillo et al., 2016; Tsarouchas et al., 2018). In response to infection, neutrophils often have roles in controlling pathogen invasive growth at later stages of infection (Gratacap et al., 2017). Neutrophils also have a role in the microenvironment of transformed cells, including glioblastoma cells, promoting their proliferation (Powell et al., 2018). Il1b, interleukin 1 beta; LC3, microtubule-associated protein light chain 3; Tnfa, tumor necrosis factor alpha; Vegfa: vascular endothelial growth factor A.

The rest of this Review will focus on innate immune cell depletion techniques in the larval zebrafish model system (Table 1, Fig. 1) and highlight some of the most recent advances from this system in the understanding of cell type-specific contributions to innate immunity in the context of infection, cancer and tissue repair (Fig. 2).

### Macrophages

Macrophages are the first immune cell to develop in zebrafish larvae, with primitive macrophages developing as early as 22 h postfertilization (Masud et al., 2017; Herbomel et al., 1999, 2001). Methods that target macrophage developmental pathways prevent the generation of these cells. Moderate knockdown of *pu.1* with a low-dose morpholino (Box 3) (Tenor et al., 2015; Rhodes et al., 2005), and morpholinos against or mutations in *irf8* (Li et al., 2011; Shiau et al., 2015), inhibit macrophage development. However, with *irf8* knockdown, the cells that would have become macrophages are diverted towards the neutrophil lineage, effectively changing the ratio of neutrophils to macrophages instead of simply depleting macrophages (Li et al., 2011; Shiau et al., 2015). Although this can complicate the analysis of the macrophages' contribution to a phenotype, in theory, it can demonstrate that even increased neutrophil numbers cannot compensate for lack of a macrophage-mediated function.

While research shows that morpholino-induced phenotypes can be the result of off-target effects (Robu et al., 2007; Gentsch et al., 2018; Kok et al., 2015), fully established and validated morpholinos are generally accepted (Stainier et al., 2017). They offer the ability to knock down gene expression in any zebrafish line without the maintenance of a transgenic or mutant background, but they do not offer much temporal control or long-term knockdown. To temporally modulate the number of macrophages in zebrafish larvae, two different methods are used: clodronate liposome injection or the nitroreductase (NTR) system. As the NTR system can be applied to a variety of cell types, it is discussed separately below.

Systemic intravenous or local clodronate liposome injection was first established as a method for macrophage depletion in mice, discussed above (Van Rooijen and Sanders, 1994), but is also applied to larval zebrafish (Bernut et al., 2014). An intravenous injection depletes macrophages throughout the body, including microglia and peripheral macrophages. Depletion of macrophages is observed as early as 6 h postinjection, although most studies perform this injection ∼24 h prior to further experimentation. Macrophage depletion can last for at least 72 h, with no effect on neutrophil numbers (Bojarczuk et al., 2016). A control injection of phosphate-buffered saline-containing liposomes accounts for the effect of the injection, which itself is an injury. A major advantage of this method is that depletion can be timed during specific periods of interest. However, one concern is that this depletion strategy is based on killing already existing cells, leaving behind dead cells that may activate immune signaling pathways (Zitvogel et al., 2010).

### Microglia

Microglia are specialized macrophages that reside in brain tissue, where they play a major role in maintaining brain homeostasis and are arguably more sensitive to changes in their environment than other tissue-resident macrophages (Gehrmann et al., 1995). However, because of their similarities to macrophages, macrophage depletion strategies also deplete microglia. Larval microglia originate not from the caudal hematopoietic tissue (CHT) and definitive hematopoeisis, but instead from early-arising cells around the yolk, in the rostral blood island (RBI) (Herbomel et al., 1999; Xu et al., 2015). One of the first innate immune-deficient zebrafish strains, *panther*, is a *csf1ra* (Box 3) mutant, and in these larvae, primitive macrophages largely fail to migrate from the RBI to colonize tissues, including the brain, resulting in larvae that are microglia deficient (Herbomel et al., 2001). These early cells in the RBI express Csf1ra and their colonization is directed by a source of the Csf1ra ligand Il34 in the brain (Wu et al., 2018; Kuil et al., 2019). *il34* mutants therefore also have fewer microglia (Wu et al., 2018; Kuil et al., 2019). Some tissue-resident macrophages also come from these early-arising cells, and *csf1ra*^−/−^ or *il34* crispant (Box 3) larvae have fewer peripheral macrophages with fewer protrusions that respond poorly to tail fin injury (Kuil et al., 2019; Wu et al., 2018; Pagán et al., 2015; Morales and Allende, 2019).

### Neutrophils

As with macrophages, targeting the development pathways of neutrophils can be an effective depletion strategy. Morpholinos against *csf3r*, which is the central controller of neutrophil development, inhibit the generation of neutrophils, but macrophage populations can also be affected (Liongue et al., 2009). There is also interest in the role of Csf3r in emergency granulopoiesis (Box 3), and while this receptor can be depleted to study emergency granulopoiesis (Willis et al., 2018; Hall et al., 2012), this pathway has roles in both basal and induced modes of neutrophil production (Liongue et al., 2009).

Other effective models to study neutrophil-dependent functions do not necessarily decrease neutrophil numbers, but instead prevent neutrophil migration from hematopoietic tissues to sites of damage and inflammation, and therefore can still be used as models of neutrophil deficiency in response to inflammatory stimuli. In some cases, blocking the main signals that govern neutrophil recruitment, Cxcl8 and Cxcr1/2 (Box 3), either through genetic manipulation or drug inhibition, is sufficient to prevent neutrophil recruitment (de Oliveira et al., 2013; Powell et al., 2018). Two transgenic lines that exemplify the strategy of modulating neutrophil motility are a zebrafish model of the rare congenital warts, hypogammaglobulinemia, immunodeficiency and myelokathexis (WHIM) syndrome [Tg(*mpx:cxcr4b^WHIM^*)] (Walters et al., 2010) and a zebrafish strain expressing a dominant-negative mutant of Rac2 [Tg(*mpx:rac2^D57N^*)] (Deng et al., 2011). In normal neutrophil development, these cells are held in the CHT by Cxcl12 (also known as SDF-1 or Cxcl12a), which signals through Cxcr4. As neutrophils mature, Cxcr4 is internalized, allowing their release from the hematopoietic tissue. Cxcr4^WHIM^ is a truncated mutant form that cannot be internalized, resulting in a persistent retention signal. The Rac2^D57N^ dominant-negative mutation directly targets neutrophil motility. Rac2 is a small GTPase that coordinates many cellular functions, including actin polymerization required for directed cell migration. Rac2^D57N^ cannot bind guanosine triphosphate (GTP) and monopolizes guanine nucleotide exchange factors required for the Rac GTPase cycle (Williams et al., 2000). Experiments with both of these lines have demonstrated that they are deficient in neutrophil activity, validating their use as models to interrogate neutrophil function (Yang et al., 2012; Gratacap et al., 2017).

### NTR system

Perhaps the most popular technique to deplete specific cell types in zebrafish is the NTR system (Curado et al., 2008). First, a bacterial NTR transgene is expressed in a cell population of choice using a cell type-specific promoter. The non-toxic pro-drug metronidazole (MTZ) is then given to and taken up by larvae through bath immersion. NTR reduces MTZ into a DNA cross-linking agent, leading to DNA damage and cell death specifically in the cells in which NTR is expressed. In zebrafish larvae, this system was first applied to heart, pancreas and liver cells (Curado et al., 2007; Pisharath et al., 2007), but has since been expanded to other cell types, including macrophages (Gray et al., 2011; Petrie et al., 2014) and neutrophils (Prajsnar et al., 2012). It was also combined with the GAL4-UAS system (Box 3) for easy interchange of other cell-specific promoters, such as *mpeg1.1* and *mpx* for macrophages and neutrophils, respectively (Box 1) (Davison et al., 2007; Ellett et al., 2011; Mathias et al., 2006).

This system has similar issues and advantages as clodronate liposomes, discussed above. MTZ treatment of these cells also causes cell death, possibly leading to immune activation. Temporal control of depletion is one of the main advantages of the NTR system. MTZ can be added at any time during experimentation to test the effect of depletion at different stages of the immune response. The duration of treatment required for full target cell population ablation varies depending on the experimental system, with most studies initiating treatment at least a day before analysis. It should also be noted that MTZ may have NTR-independent effects on some phenotypes of interest (Oehlers et al., 2015) and MTZ treatment of non-NTR larvae should always be included as a control condition.

## Recent findings made possible by these models

The innate immune depletion models in larval zebrafish discussed above can be used to investigate the role of macrophages and neutrophils in a variety of immune contexts including infection, wounding and cancer. Here, we highlight a few recent studies (Fig. 2).

### Macrophages as controllers of infection

The role of macrophages in the innate immune system's response to infection is different for each infection context. Macrophages are required for full control of the bacterial pathogen *Salmonella* (Masud et al., 2019). Using the live-imaging capabilities of larval zebrafish, Masud et al. (2019) found that LC3-associated phagocytosis by macrophages promotes control of this pathogen. NTR-mediated macrophage depletion resulted in 100% larval death by 24 h postinfection and increased bacterial replication, results that were confirmed with an anti-*irf8* morpholino. Neutrophil depletion with the NTR system also somewhat increased bacterial burden, but it was clear that macrophages play a larger role in the early control of this infection.

Two other recent papers also reported a role for macrophages in controlling the growth of a pathogen, in this case the fungal pathogen *Cryptococcus neoformans* (Box 3) (Tenor et al., 2015; Bojarczuk et al., 2016). Both studies injected zebrafish larvae with the yeast form of a highly virulent strain of *C. neoformans*, H99. Although the yeast could in some cases replicate inside of macrophages, removal of macrophages with either low-dose anti-*pu.1* morpholino (Tenor et al., 2015) or clodronate liposomes (Bojarczuk et al., 2016) decreased the survival of larvae and increased fungal growth. Bojarczuk et al. (2016) also took advantage of the temporal control offered by clodronate liposome injection, demonstrating that removal of macrophages after an infection is established still leads to an increased fungal burden.

### Macrophages as a protective niche for pathogens

Despite their importance in fighting infection, macrophages do not control growth of all pathogens and can, for some infections, serve as a protective or proliferative niche. By residing in macrophages, pathogens are protected from recognition and destruction by both soluble factors such as the complement system and by other immune cells. Macrophages can be a protective niche for bacteria as well, and Mesureur et al. (2017) recently investigated this question in the context of *Burkholderia cenocepacia* infection. Using either low-dose anti-*pu.1* morpholino or the NTR system, this study found that larvae without macrophages had increased survival after infection. Although bacteria could replicate in the absence of macrophages, the presence of macrophages increased bacterial growth. Similar neutrophil depletion experiments with the NTR system or a *csf3r* morpholino had no effect on larval survival, again demonstrating a specific role for macrophages as a proliferative niche (Mesureur et al., 2017).

A different study of infection with the fungal pathogen *C. neoformans* found that when zebrafish larvae are infected with a less virulent strain and with spores instead of the yeast form of the pathogen, macrophages can play a pathogen-protective role (Davis et al., 2016). These fungal spores are phagocytosed by macrophages but later escape back into the vasculature. Removal of this early intracellular niche in an *irf8* mutant actually resulted in a lower fungal burden, although the increased neutrophil numbers in this genetic background may also contribute to fungal clearance (Davis et al., 2016).

Two other studies came to similar conclusions with fungal pathogens recently, using multiple models of macrophage depletion (Rosowski et al., 2018a; Ellett et al., 2018). Rosowski et al. (2018a) reported that macrophage deficiency, in either an *irf8* mutant or upon clodronate liposome injection, led to increased clearance of a fast-growing strain of the fungus *A. fumigatus*, but not of a slower-growing strain. The ability of macrophages to inhibit fungal germination and growth actually inhibited neutrophil-mediated killing of the faster-growing strain. In a *Taloromyces marneffei* fungal infection, the infectious burden also decreased when macrophages were removed, either by anti-*irf8* morpholino or by the NTR system (Ellett et al., 2018). Conversely, neutrophil depletion, either through anti-*csf3r* morpholino or the NTR system, had little effect on fungal burden. In fact, depletion of both cell types with a combination of anti-*pu.1* and anti-*csf3r* morpholinos also decreased early fungal burden, underlining the importance of the macrophage intracellular niche for fungal growth (Ellett et al., 2018).

### Macrophages promote vascular repair

The role of macrophages in the repair of blood vessels has become an area of active research in larval zebrafish, finding that macrophages can mediate vascular repair (Liu et al., 2016; Gurevich et al., 2018; Gerri et al., 2017). Mechanical or laser-mediated damage to blood vessels in either the brain or the tail recruits macrophages to the injury site, where they wrap around or adhere to the wounded vessels (Liu et al., 2016; Gurevich et al., 2018). Knockdown of macrophages with anti-*irf8* morpholino resulted in deficient blood vessel repair in the brain (Liu et al., 2016), while depletion with either the NTR system or clodronate liposomes resulted in a failure of tail fin vessel repair (Gurevich et al., 2018). *Csrf1ra^−/−^* larvae also have impaired healing of the tail vasculature, suggesting that peripheral macrophages in particular promote this repair (Gurevich et al., 2018). By temporally controlling macrophage depletion with administration of MTZ or clodronate liposomes after initial vessel repair already occurred, Gurevich et al. (2018) also implicated macrophages in vessel pruning at later stages of regeneration.

How do macrophages mediate this repair and regrowth? In the case of targeted brain blood vessel damage with a multi-photon laser, a single macrophage is often recruited to the injury site and mediates repair through mechanical forces, adhering to the ruptured vessel ends and pulling them together (Liu et al., 2016). Interestingly, if the first macrophage to arrive to the injury site is also laser-ablated, this loss impairs repair; although another macrophage is recruited to phagocytose the dead cell, this second macrophage does not engage with the injured vessel (Liu et al., 2016). Several studies also implicate Vegfa (also known as Vegfaa; Box 3) in macrophage-mediated angiogenesis and repair (Gurevich et al., 2018; Oehlers et al., 2015; Britto et al., 2018).

### Macrophages increase repair of damaged nerve tissue

Larval zebrafish can effectively repair and regenerate nerve cells, and macrophages have been implicated in this process. Two recent studies demonstrated that, after nerve damage, both neutrophils and macrophages are recruited to the injury site, with neutrophil numbers peaking early and then resolving away, whereas macrophages are more persistent at the wound (Carrillo et al., 2016; Tsarouchas et al., 2018). In a model of mechanosensory hair cell (Box 3) damage caused by exposure of lateral line hair cells to copper, macrophage depletion by either low-dose anti-*pu.1* morpholino or local clodronate liposome injection delays regeneration of these hair cells (Carrillo et al., 2016). Larval zebrafish can repair spinal cord injuries, including complete spinal cord transection. In a transection model, Tsarouchas et al. (2018) found that altering the level of overall inflammation by treatment with a glucocorticoid drug or lipopolysaccharide (LPS; Box 3) modulates regeneration, with increased immune cell recruitment associated with better outcome. Using an *irf8* mutant, they found that macrophages are not required for the initial repair steps but are required for complete recovery of spinal cord function, as measured by larval swimming. *Csf1ra^−/−^* larvae do not have defects in this repair, suggesting that this regeneration function of macrophages is due to recruited macrophages, not tissue-resident cells or microglia (Tsarouchas et al., 2018).

### Macrophages regulate the inflammatory environment at sterile wounds

Multiple recent papers have focused on the role of macrophages in modulating the immune environment at an injury site, especially through regulation of the pro-inflammatory cytokines *il1b* and *tnfa* (Box 3) (Tsarouchas et al., 2018; Morales and Allende, 2019; Nguyen-Chi et al., 2017; Hasegawa et al., 2017). These pro-inflammatory mediators are turned on early in the wound response and downregulated in later stages of repair (Tsarouchas et al., 2018; Hasegawa et al., 2017). In contrast, in macrophage-deficient zebrafish, this early expression is impaired but increases later. Morpholino-mediated knockdown or drug inhibition of pro-inflammatory cytokine expression impairs regeneration in wild-type larvae, but improves repair in macrophage-deficient ones (Hasegawa et al., 2017; Nguyen-Chi et al., 2017). Overall, these studies suggest that although early expression of these pro-inflammatory genes is required for efficient repair, their expression must be downregulated to promote late healing and implicate macrophages in both early and late healing phases (Tsarouchas et al., 2018; Hasegawa et al., 2017; Nguyen-Chi et al., 2017). Work in a *csf1ra* mutant identified peripheral macrophages as the cells responsible for downregulating *il1b* expression (Morales and Allende, 2019). One function of *tnfa* signaling may be to support the proliferation of cells in the blastema (Box 3) (Nguyen-Chi et al., 2017). Using a *tnfa* expression reporter, Nguyen-Chi et al. (2015) identified macrophages as sources of *tnfa* at the wound site, with early macrophages expressing *tnfa* when first responding to the injury and then converting to a *tnfa*-negative phenotype later in repair. This group then used parabiosis (Box 3) experiments to confirm that macrophage-produced Tnfa can signal to stromal cells to support the proliferation of blastemal cells (Nguyen-Chi et al., 2017).

However, there are two major confounding factors that could alter the interpretation of these macrophage-depletion experiments and the assignment of specific roles to macrophages in wound repair. First, the presence of neutrophils at a wound may be increased in macrophage-deficient larvae, and, as neutrophils can cause tissue damage, an increased presence of neutrophils may be the factor that delays repair and regeneration. This is especially true for experiments with *irf8* mutants or morphants that have increased total numbers of neutrophils, but may also occur with other macrophage depletion methods, as macrophages can promote neutrophil resolution from wounds (Tauzin et al., 2014; Loynes et al., 2018). In a blood vessel repair model, more neutrophils were observed at the injury site after macrophage depletion (Gurevich et al., 2018). Additionally, in a spinal cord injury model, neutrophils were identified as a major producer of *il1b*, and depletion of neutrophils with anti-*pu.1* and anti-*csf3r* morpholinos in *irf8* mutant zebrafish larvae improved repair when compared to *irf8* mutation alone (Tsarouchas et al., 2018).

A second issue is that wounding leads to a significant level of cell death, and a major role of macrophages is to phagocytose dead cells and debris (Tabas, 2010). Several studies documented an increase in the number of dead cells at the wound and in the blastema after tail fin amputation in macrophage-deficient larvae (Hasegawa et al., 2015, 2017; Loynes et al., 2018; Morales and Allende, 2019). However, the interpretation of this observation has differed, with studies concluding either that macrophages produce a signal to promote the survival of these cells (Hasegawa et al., 2015, 2017) or that these are cells that would normally be cleared by phagocytic macrophages (Loynes et al., 2018). It is also unclear what the effect is of these dead cells on *tnfa*, *il1b* and other pro-inflammatory cytokine levels, as it is possible that their very appearance or failure to be removed activates inflammatory pathways (Zitvogel et al., 2010). The role of phagocytosis in these phenotypes was addressed in the context of spinal cord injury, where chemical inhibition of phagocytosis alone did not impair regeneration (Tsarouchas et al., 2018), but remains unclear in other models.

### Neutrophils control invasive infection

Neutrophils are often the first responders to infection or tissue damage, and some infections are characterized by early neutrophil recruitment and neutrophil-mediated immunity (Rosowski et al., 2016; Willis et al., 2018). However, in other cases, such as mycobacterial infection (Yang et al., 2012) or infections with the fungal pathogens *A. fumigatus* (Rosowski et al., 2018a) or *C. neoformans* (Davis et al., 2016), neutrophils arrive later and control late-stage infection, combating invasive growth of the pathogen.

A recent study by Gratacap et al. (2017) illustrates this late role of neutrophils in controlling invasive growth in a model of mucosal candidiasis in zebrafish. Modulating neutrophil activity at the infection site with two different methods, Tg(*mpx:rac2^D57N^*) and chemical inhibition of Cxcr2, resulted in increased larval host death. Rac2^D57N^ neutrophil-defective larvae have increased fungal filamentation, leading to increased damage to the epithelial barrier surrounding the infection site, suggesting that neutrophils control this later, invasive growth stage of infection (Gratacap et al., 2017).

### Neutrophils promote transformed cell proliferation

The innate immune system also plays a role in the response to transformed cells and cancer, with neutrophils having both pro- and anti-tumor functions (Giese et al., 2019). Neutrophils promote early stages of cancer progression in multiple transformed cell models in larval zebrafish (Freisinger and Huttenlocher, 2014; Feng et al., 2012). In fact, increasing neutrophil recruitment to a developing clone of transformed cells by creating a nearby tissue wound increases the proliferation of that clone (Antonio et al., 2015). Powell et al. (2018) recently expanded these studies to a model of glioblastoma with Kras-transformed astrocytes. In Rac2^D57N^ neutrophil-defective larvae, Kras^+^ cells are less proliferative. Additionally, *cxcr1^−/−^* larvae or larvae treated with a Cxcr1/2 inhibitor had decreased neutrophil recruitment to and proliferation of Kras^+^ cells, identifying a major signaling axis for neutrophil stimulation of transformed cell proliferation (Powell et al., 2018).

## Future directions

### Innate immune cell subsets

The models discussed above were developed to deplete entire cell populations, either neutrophils, macrophages or tissue-resident macrophages such as microglia, but these cell types exist in a variety of activation states (Murray et al., 2014; Silvestre-Roig et al., 2019), and one future direction will be to determine the roles of the individual cell subsets through subset-specific depletion strategies. One categorization of macrophage subtypes relies on anatomical source – tissue-resident versus recruited (Box 1). Mutation of *csf1ra* is one method to modulate peripheral macrophages specifically, including microglia as discussed above, but it is still unclear what percentage of non-microglial peripheral macrophages depend on the Csf1ra pathway for their localization (Xu et al., 2015; Herbomel et al., 2001). The Ramakrishnan laboratory has also delineated separate functions of tissue-resident and infiltrating macrophages in the response to mycobacterial infection (Cambier et al., 2014, 2017). They find that *Mycobacterium marinum* escape killing by tissue-resident macrophages, and instead recruit fewer microbicidal Ccr2^+^ monocytes. The recruitment of these permissive monocytes and their function at the infection site can be inhibited by morpholinos against either *ccr2* or its ligand, *ccl2* (Cambier et al., 2014), but the role of the Ccl2–Ccr2 signaling axis in other inflammatory responses is unknown. The main marker of macrophage transcriptional polarization used in zebrafish has been *tnfa* expression (Nguyen-Chi et al., 2015). Either *tnfa* or *tnfr1* morpholinos can block this signaling, but it remains unclear how this knockdown affects overall macrophage polarization or the behavior of other immune cells (Nguyen-Chi et al., 2017).

### Disease-specific deficiency models

Larval zebrafish have emerged as models of a variety of human genetic diseases. In fact, two of the neutrophil-defective models discussed here, the WHIM and Rac2^D57N^ models, were developed as models for human disease mutations (Walters et al., 2010; Deng et al., 2011). Although most work on innate immune cell function in zebrafish has focused on the role of macrophages and neutrophils in wild-type larvae, the zebrafish presents a highly useful model system in which to understand the requirement for (and defective phenotypes of) these cells in the context of other immune deficiencies. Two such genetic diseases that already have zebrafish models are cystic fibrosis (Bernut et al., 2019) and phagocyte oxidase deficiency (Tauzin et al., 2014), the cause of chronic granulomatous disease. Another disease factor in humans, high-fat diet, was recently modeled in larval zebrafish in the context of liver cancer (de Oliveira et al., 2019). Here, macrophages promoted hepatocellular carcinoma progression specifically in animals that were fed a high-fat diet, highlighting the importance of combining these immune deficiency models with other disease factors to fully understand disease mechanisms and identify therapeutic opportunities (de Oliveira et al., 2019).

### Innate immune cell reconstitution with mammalian cells

Immune depletion models in fish will also allow researchers to reconstitute the immune system with cells derived from humans or mammalian models, in order to directly visualize the behavior of these cells in complex tissues, similar to ‘humanized’ mouse models (Ito et al., 2018). Mouse neutrophils co-injected with *C. albicans* were at least partially functional as they could somewhat decrease fungal burden in Rac2^D57N^ neutrophil-defective larvae (Gratacap et al., 2017). Transplantation of murine bone marrow cells (Parada-Kusz et al., 2018), human hematopoietic stem cells (Hamilton et al., 2018) and human macrophages (Paul et al., 2019) into zebrafish is possible but has not yet been applied to studies of the behavior of these cells in response to infection or injury.

## Conclusions

Determining the specific requirements and functions of different innate immune cells in response to insults such as infection, wounding and cancer is key for future development and implementation of patient treatments. Knowing how different cell types' activities improve or worsen disease progression is paramount for deciding whether to use treatments that seek to modulate the numbers of a given immune cell type, such as Csf3 administration to increase neutrophil production, and for identifying new molecular targets that modulate innate immune cell activity. As discussed in this Review, the function of each of these cells can vary greatly in each disease context. While much continues to be learned from the mouse model, the expansion of cell depletion models and live imaging methods in larval zebrafish make this animal model a fruitful system for research on innate immune function.
